# Treatment experience of delayed massive gastrointestinal bleeding caused by intra-abdominal arteriointestinal fistula in gastric cancer patients after radical gastrectomy

**DOI:** 10.1186/s12957-019-1751-0

**Published:** 2019-11-30

**Authors:** Liang Chen, Xuezhi Ming, Rongmin Gu, Xu Wen, Gang Li, Bin Zhou, Wei Wei, Huanqiu Chen

**Affiliations:** Department of General Surgery, Jiangsu Cancer Hospital & Jiangsu Institute of Cancer Research & The Affiliated Cancer Hospital of Nanjing Medical University, No. 42, Baiziting, Nanjing, Jiangsu Province, 210009 China

**Keywords:** Gastric cancer, After radical gastrectomy, Arteriointestinal fistula, Delayed massive gastrointestinal bleeding, Treatment

## Abstract

**Background:**

Gastric cancer (GC) remains one of the leading causes of cancer-related death. Arteriointestinal fistula is a very rare but lethal postoperative complication in GC patients after gastrectomy. However, very few reports associated with arteriointestinal fistula have been published, and there is no matured diagnosis and treatment consensus for arteriointestinal fistula. Herein, we will investigate the etiology, clinical feature, diagnostic method, treatment, and prognosis by summarizing two patients we treated and consulting related cases reported in recent years.

**Case presentation:**

A 61-year-old male and 75-year-old female with advanced gastric cancer of gastric antrum underwent radical distal gastrectomy and D2 regional lymphadenectomy. Residual gastrojejunostomies by the Roux-en-Y method were performed. The two patients recovered well after gastrectomy, and they received postoperative adjuvant chemotherapy. However, both of them suffered sudden hematemesis and melena about 2 months after surgery, resulting in unstable vital signs. Emergency exploratory laparotomy and interventional embolotherapy by digital subtraction angiography were immediately respectively performed. During this process, arteriointestinal fistulas were found in both of them. Pseudoaneurysms of gastroduodenal artery and common hepatic artery were respectively ruptured and bleeding into the duodenum. Finally, the male patient recovered, while the female patient died because of rebleeding and hemorrhagic shock.

**Conclusions:**

Arteriointestinal fistula, with low morbidity but high mortality, is an acute and fatal postoperative complication for GC patients after radical gastrectomy. DSA is the preferred method to diagnose arteriointestinal fistula. Embolotherapy by DSA should be performed immediately once arteriointestinal fistula is confirmed. Emergency laparotomy is another selection if the embolotherapy failed. We should pay more attention to perioperative preventive measures for formation of pseudoaneurysm, which is the leading cause of arteriointestinal fistula.

## Background

Gastric cancer (GC) is a frequently lethal malignancy that is the fifth most commonly diagnosed cancer and the third leading cause of cancer-related death [1]. GC is particularly common in eastern Asia [1]. The prognosis of GC is poor, especially for advanced GC patients.

Surgical resection remains the main treatment for GC. Postoperative lethal complications are the most important challenges for the surgeon, among which pseudoaneurysm caused by intraoperative or postoperative vascular wall injury is a rare complication [2]. The incidence of pseudoaneurysm has increased in recent years and it has become an important factor for influencing the safety of radical gastrectomy [3]. Arteriointestinal fistula resulting from rupture of the pseudoaneurysm into the gastrointestinal tract is a very rare but lethal postoperative complication in GC patients after gastrectomy. In abdominal surgery, arteriointestinal fistula has very low morbidity but high mortality because of its burstiness [4]. However, the pathogenesis of arteriointestinal fistula is not entirely clear and requires further attention.

Very few reports have described arteriointestinal fistula in GC patients after radical gastrectomy. Furthermore, consensus on the diagnosis and treatment of arteriointestinal fistula is lacking. Herein, we present two cases of delayed massive upper gastrointestinal bleeding in patients who had previously undergone radical gastrectomy in our hospital. The aim of this study is to analyze etiology, clinical features, diagnostic methods, treatment, and prognosis by summarizing these two cases and consulting related cases reported in recent years.

## Methods

### Case collection

The details of the cases were collected from two GC patients with delayed massive upper gastrointestinal bleeding resulting from arteriointestinal fistula after radical gastrectomies performed in the general surgery department of the affiliated cancer hospital of Nanjing Medical University in February 2016 and March 2019, respectively. Relatives of the two patients were informed of the study and signed informed consent. We retrospectively analyzed the data on clinical features, diagnosis, treatment, and prognosis. The average age was 68 years old (61 and 75 years, respectively). Although embolotherapy was temporarily effective, one patient died because of recurrent massive upper gastrointestinal bleeding. And, one patient was cured by emergency laparotomy and long-time recovery because of postoperative complications; no bleeding reoccurred during the following 24 months of follow-up.

### Literature review

Several keywords (including gastric cancer, pseudoaneurysm, upper gastrointestinal bleeding, and arteriointestinal fistula) were used to consult literatures in Pubmed and China National Knowledge Infrastructure (CNKI) databases. We identified 12 cases of arteriointestinal fistula after radical gastrectomy in the literature. All presented as sudden onset of massive upper gastrointestinal bleeding caused by arteriointestinal fistula but with differences in arteriorrhexis and/or the site of gastrointestinal fistula. Laparotomy and/or embolotherapy was performed immediately for all patients. Only 8 patients were cured finally, and 4 patients died because of hypovolemic shock.

## Results

### Case 1

A 61-year-old male was admitted to our hospital 2 months after radical gastrectomy for “sudden hematemesis and black stool for 1 day.” He had previously been diagnosed with gastric cancer and underwent radical distal gastrectomy, Roux-en-Y anastomosis, and D2 regional lymphadenectomy on 16 February 2016. The postoperative pathological outcomes were as follows: the tumor was medium-to-low differentiated tubular adenocarcinoma in the lesser curvature of the gastric antrum, Lauren’s type was mixed type, the tumor cells involved the whole layer of gastric wall and had reached the adipose connective tissue, the nerve was invaded, and no metastasis was observed in all perigastric lymph nodes. The patient recovered well after gastrectomy. He received two cycles of chemotherapy with Oxaliplatin (200 mg, day 1) and tegafur-gimeracil-oteracil potassium (50 mg, bid, day 1 to day 14) on 11 March 2016 and 29 March 2016.

Two months after surgery, the patient presented with sudden hematemesis, tarry stools, and brief loss of consciousness on 16 April 2016. Fast blood transfusion, fluid infusion, and hemostatic therapy did not relieve the symptoms. The patient’s blood pressure rapidly dropped to 63/46 mmHg because of persistent bleeding, and his heart rate reached 160 beats per minute. Therefore, emergency exploratory laparotomy was performed. The outcomes of intraoperative exploration were as follows: a large amount of clear ascites was observed in the abdominal cavity, and the small intestine and colon were markedly dilated, with abundant hematoceles in the small intestine. Blocking the input loop and output loop using bowel forceps resulted in increased tension in the input loop, indicating that the bleeding site was in the duodenum. Massive bleeding and common bile duct injury appeared during the tissue separation process because of cohesive and dense tissue around the duodenal stump. Finally, the patient’s vital signs recovered smoothly after ligating the gastroduodenal artery (GDA). Several drainage tubes were placed in the abdominal cavity at the end of surgery.

The patient presented with intestinal fistula and biliary fistula postoperatively. After long-term treatment with best supportive care including fasting, fluid infusion, hemostasis, and nutritional support, the patient was discharged from the hospital upon recovery on 13 September 2016 (5 months after emergency surgery). No bleeding reoccurred within 24 months of follow-up.

### Case 2

A 75-year-old female was admitted to our hospital more than 2 months after radical gastrectomy because of “sudden hematemesis and tarry stool for 1 day.” She had undergone radical distal gastrectomy, Roux-en-Y anastomosis, and D2 regional lymphadenectomy on 5 March 2019 after a diagnosis of gastric cancer. The postoperative pathological results were as follows: the tumor was a low differentiated adenocarcinoma with an ulcer in the gastric antrum, Lauren’s type was mixed type, the tumor cells involved the whole layer of gastric wall and had reached to the adipose connective tissue, the tumor tended to be hepatoid adenocarcinoma, and all perigastric lymph nodes were confirmed to have no metastasis. The patient recovered well. She received one cycle of chemotherapy of oxaliplatin (100 mg, day 1) and tegafur-gimeracil-oteracil potassium (50 mg, bid, day 1 to day 14) on 16 April 2019. The patient did not undergo additional cycles of chemotherapy due an intolerance of severe side effects such as asitia, nausea, and epigastric discomfort.

**Fig. 1:**
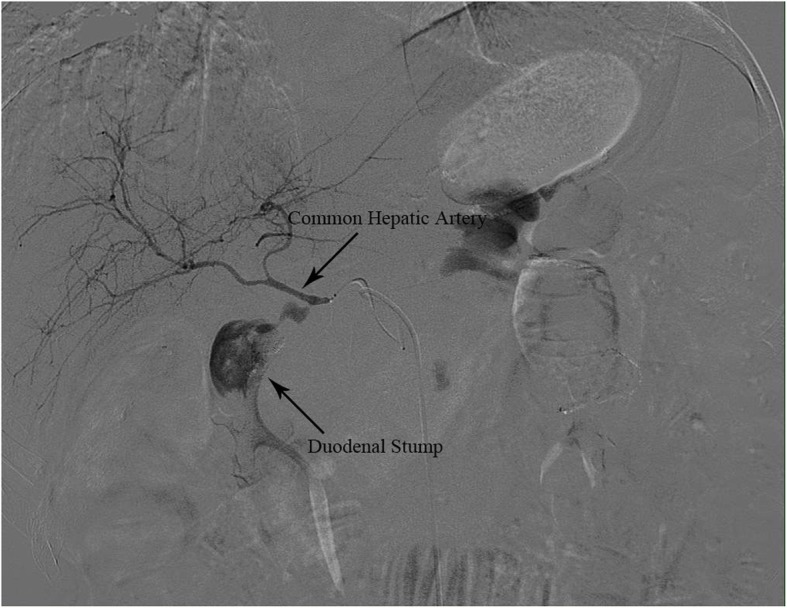
Aneurysm of common hepatic artery was ruptured and bleeding into the duodenal stump

Sixty-three days after radical gastrectomy, the patient was emergency admitted to our hospital on 7 May 2019 due to sudden hematemesis and tarry stools. Her symptoms were relieved to some extent after immediate treatment with blood transfusion, fluid infusion, and hemostatic therapy. However, the patient subsequently vomited blood again several times, with a total volume of 800 ml, and experienced tarry stools with a total volume of approximately 1000 ml. Moreover, her blood pressure rapidly dropped to 60/40 mmHg, and her heart rate reached 130 beats per minute. Emergency digital subtraction angiography (DSA) was performed to find the bleeding site and revealed that an aneurysm of the common hepatic artery (CHA) had ruptured and was bleeding into the duodenal stump (Fig. 1). Embolization of the hepatic artery as an intervention for hemostasis resulted in gradual stabilization of the patient’s vital signs.

However, after 2 hours, sudden hematemesis and tarry stools appeared again. Rebleeding of the CHA was considered, and the relatives of the patient were informed of the need for second embolotherapy or exploratory laparotomy. However, the relatives requested conservative treatment. Despite active efforts to increase the patient’s blood pressure using dopamine, blood transfusion, fluid infusion, and hemostasis, hemorrhagic shock ultimately occurred. The patient subsequently died after against-advice discharge.

## Clinical characteristics of 12 patients in literature review

In 10 cases (83.3%), the patient was male; gender was not indicated in 2 cases (16.7%). Of the 10 cases (83.3%) for which age was reported, the average age was 61.6 years (range from 35 to 76 years). Radical distal gastrectomy was performed in 9 cases (75%, including Roux-en-Y anastomosis in 2 cases, Billroth II anastomosis in 6 cases and not indicated in 1 case), and radical total gastrectomy and Roux-en-Y anastomosis were performed in 3 cases (25%). The average time from radical gastrectomy to bleeding was 37.9 days (range from 25 to 67 days). The bleeding artery was the GDA in 4 cases (33.3%), CHA in 4 cases (33.3%), splenic artery (SA) in 2 cases (16.7%), proper hepatic artery (PHA) in 1 case (8.3%), and right hepatic artery (RHA) in 1 case (8.3%). The location of the arteriointestinal fistula was in the duodenum in 9 cases (75%), jejunum loop in 2 cases (16.7%), and remnant stomach in 1 case (8.3%). Two cases (16.7%) were diagnosed by exploratory laparotomy, and 10 cases (83.3%) were diagnosed by DSA. Although exploratory laparotomy and/or embolotherapy were actively performed, only 8 patients were ultimately cured, and 4 patients died from hypovolemic shock, corresponding to a mortality rate of 33.3% (Table 1).

**Table 1:** Clinical characteristics of 12 patients with delayed arteriointestinal fistula after radical gastrectomy in literature review

Case number	Gender	Age (years)	Operation type	Bleeding time (days)	Clinical feature	Bleeding artery	Site of fistula	Diagnostic method	Treatment method	Prognosis
1^a^	Male	35	DG/B II	46	Hematemesis, ventosity, shock	Common hepatic artery	Duodenum	DSA	Embolism	Died
2^a^	Male	76	DG/R-Y	37	Tarry stool, hematemesis, shock	Gastroduodenal artery	Duodenum	DSA	Embolism	Cured
3^a^	Male	62	DG/B II	25	Hematemesis, ventosity	Common hepatic artery	Duodenum	DSA	Embolism, operation	Died
4^a^	Male	56	DG/B II	30	Hematemesis, abdominal pain	Proper hepatic artery	Duodenum	DSA	Operation	Cured
5^a^	Male	70	DG/B II	26	Hematemesis, ventosity	Gastroduodenal artery	Duodenum	DSA	Operation	Died
6^b^	Male	64	DG/-	30	Tarry stool, hematemesis, shock	Common hepatic artery	Remnant stomach	DSA	Embolism	Died
7^c^	Male	52	TG/R-Y	35	Hematemesis, abdominal pain	Splenic artery	Jejunum loop	DSA	Embolism	Cured
8^c^	Male	61	TG/R-Y	37	Tarry stool, hematemesis, shock	Splenic artery	Jejunum loop	DSA	Embolism	Cured
9^c^	Male	70	TG/R-Y	65	Tarry stool, hematemesis	Common hepatic artery	Duodenum	DSA	Embolism	Cured
10^c^	Male	70	DG/R-Y	67	Fever, tarry stool, abdominal pain	Right hepatic artery	Duodenum	DSA	Embolism	Cured
11^d^	-	-	DG/B II	27	Tarry stool, hematemesis	Gastroduodenal artery	Duodenum	Exploratory laparotomy	Operation, embolism	Cured
12^d^	-	-	DG/B II	30	Tarry stool, hematemesis	Gastroduodenal artery	Duodenum	Exploratory laparotomy	Operation, embolism	Cured

*DG* distal gastrectomy, *TG* total gastrectomy, *R-Y* Roux-en-Y anastomosis, *B II* Billroth II anastomosis, *DSA* digital subtraction angiography

^a^Five patients were reported by Wang et al. [5]

^b^One patient was reported by Satoh et al., the anastomotic method was not indicated [6]

^c^Four patients were reported by Chen et al. [7]

^d^Two patients were reported by Tong et al., the gender and age of which were not indicated [8]

## Discussion

The incidence of GC, a common lethal malignancy, has increased markedly in recent years, particularly in eastern Asia [1]. Radical gastrectomy continues to be the most important therapeutic method for GC patients. As a rare but lethal postoperative complication, delayed massive gastrointestinal bleeding caused by arteriointestinal fistula after radical gastrectomy has rarely been reported in the literature. In this analysis of our practical experience and the literature, we explored the potential pathogenesis, clinical features, diagnostic methods, treatment modalities, prognosis, and preventive measures of delayed massive gastrointestinal bleeding resulting from arteriointestinal fistula in GC patients after radical gastrectomy.

Previous reports have not definitively confirmed the etiology of arteriointestinal fistula after radical gastrectomy. A variety of factors can result in pseudoaneurysm, such as infection, trauma, and surgical procedures [2]. Consequently, pseudoaneurysm formation can be caused by direct or indirect injury of the arterial wall [5]. Therefore, we propose two predominant pathways of pathogenesis of arteriointestinal fistula after radical gastrectomy.

According to one of these pathways, in patients with gastrointestinal fistula such as duodenal stump fistula, digestive juice effused from the digestive tract corrodes the arterial wall and damages its integrity. In this study, 3 patients with duodenal stump fistula or dormant duodenal stump fistula were identified in the literature [5, 8]. The synergistic role of infection and chemical corrosion by the digestive juice indirectly damages the wall of the GDA, leading to the formation of a pseudoaneurysm whose rupture ultimately results in arteriointestinal fistula.

Alternatively, pseudoaneurysm resulting from many factors after radical gastrectomy ruptured into gastrointestinal tract. Several factors, including damage to the vasculature and perivascular nerve plexus in the vascular skeletonization process during lymphadenectomy, injury of the adventitia resulting from inappropriate use of the medical electric knife and ultrasound knife, and damage to the endarterium caused by ligation using inapposite sutures or crude clamping with vascular forceps, can result in chronic formation of pseudoaneurysm [3]. Furthermore, injury of the arterial wall resulting from postoperative persistent friction between the staple lines and bare arterial wall and perioperative radiotherapy has also been reported as a cause of pseudoaneurysm formation [9–11]. In general, rupture of the pseudoaneurysm mostly leads to intra-abdominal bleeding, and rupture into the gastrointestinal tract is rarely reported [5]. Various tissues and organs of the upper abdomen may stick to each other after radical gastrectomy, and the epigastric postoperative region is a relatively closed space. Consequently, pseudoaneurysm formation could oppress the surrounding tissues or organs. We speculate that when the gastrointestinal wall or a relatively weak duodenal stump surrounds the pseudoaneurysm and is chronically oppressed by it, the gastrointestinal wall and duodenal stump tissues may become ischemic and even necrotic, leading to gastrointestinal fistula or duodenal stump fistula. Formation of these fistulas finally leads to arteriointestinal fistula and massive hemorrhage of the gastrointestinal tract, without intra-abdominal bleeding. Atherosclerosis has also been reported to be an important cause of pseudoaneurysm formation [12]. Therefore, arteriointestinal fistula is the consequence of a variety of pathological processes.

Arteriointestinal fistula can also occur rarely in patients with no history of surgery. A previous study reported a case without previous operation in which a splenic artery aneurysm ruptured into the splenic flexure of the colon, leading to massive lower gastrointestinal bleeding [13]. The clinical manifestations of arteriointestinal fistula are diverse and non-specific. According to the study described by Shen KT and the cases we collected, hematemesis, black stool, hypovolemic shock, abdominal pain, and abdominal distension are the most common symptoms of arteriointestinal fistula [3]. A previous study showed that abdominal pain, a palpable abdominal mass and gastrointestinal hemorrhage, was the classic triad of symptoms for primary aortoenteric fistula [14]. In addition, primary aortoenteric fistula exhibits distinctive gastrointestinal hemorrhage regarded as “herald” bleeding, in which a small amount of gastrointestinal bleeding appears intermittently and repeatedly prior to the appearance of fatal massive gastrointestinal bleeding [15]. This type of bleeding is thought to be associated with an initially minor fistula. The gastrointestinal bleeding stops temporarily due to thrombus in the pseudoaneurysm, intestinal contracture, and decreased arterial blood pressure. However, gastrointestinal bleeding reappears upon movement or dissolution of the thrombus and an increase in arterial blood pressure. As the fistula expands, fatal gastrointestinal bleeding will suddenly emerge. Therefore, the time period before fatal bleeding is considered the most important for emergency exploratory laparotomy. In our study, we observed this distinctive gastrointestinal hemorrhage in the second patient we managed. Unfortunately, the brief and vital time interval for emergency laparotomy had already elapsed.

The lack of distinctive symptoms of arteriointestinal fistula is a challenge for rapid diagnosis. For patients with massive gastrointestinal tract bleeding, endoscopy is not the optimal diagnostic method because of the limitation of unclear vision [5]. It is difficult to locate the fistula and provide any treatment measures via endoscopy. However, enhanced computed tomography (CT) and abdominal ultrasonography can be helpful for the diagnosis of gastrointestinal tract bleeding in patients based on the potential of these techniques to exclude intra-abdominal hemorrhage and retroperitoneal hematoma [3]. DSA should be considered as the preferred diagnostic method, especially for patients with unstable vital signs or “herald” bleeding [5, 7]. DSA can directly display the pseudoaneurysm and locate the bleeding site for subsequent embolotherapy. Interventional embolotherapy by DSA could achieve good hemostatic effects in some patients. In our analysis, 5 of 8 patients were finally cured by sole embolotherapy by DSA. For the remaining patients, embolotherapy by DSA only temporarily stopped the pseudoaneurysm hemorrhage but provided sufficient time for preparation for exploratory laparotomy.

Although interventional embolotherapy is the preferred treatment method, the cure rate is not high because of the risk of rebleeding. The cure rate in our study was 62.5%. However, postoperative adhesion of abdominal tissue, inflammatory edema, coagulopathy, and poor physical condition in patients with delayed massive hemorrhage of the gastrointestinal tract after radical gastrectomy can obviously increase the risk of exploratory laparotomy [3, 7]. Therefore, it is important to specify the indications for interventional embolotherapy or emergency laparotomy according to the condition of the patient.

As a fatal postoperative complication, the mortality rate of arteriointestinal fistula is reported to be more than 50% [2, 5]. The mortality rate in our study was 35.7%. Due to its poor prognosis, the prevention of arteriointestinal fistula requires greater attention than its treatment. First, understanding the patient’s history and preoperative staging adequately before the operation is necessary. Second, serious intraoperative consideration must be given to protecting the integrity of the adventitia, avoiding thermal injury to the adventitia caused by the medical electric knife or ultrasound knife, reducing damage to the endarterium caused by inapposite ligation or clamping, and covering all stapled edges by suturing. Finally, we must reduce the incidence of perioperative radiotherapy injury and postoperative complications, such as intra-abdominal infection, anastomotic fistula, and duodenal stump fistula, which can result in formation of pseudoaneurysm.

In conclusion, arteriointestinal fistula is an acute and lethal postoperative complication for patients after radical gastrectomy. Despite its low incidence rate, arteriointestinal fistula must be kept in mind when encountering patients with unexplained gastrointestinal hemorrhage or “herald” bleeding after previous radical gastrectomy. DSA is the preferred method for diagnosing arteriointestinal fistula. Once confirmed, interventional embolotherapy by DSA should be performed immediately to stop the arterial bleeding. If embolotherapy fails, emergency exploratory laparotomy is an option to increase the survival rate of patients. Moreover, due to its high mortality, greater attention to the prevention of arteriointestinal fistula is warranted. Avoiding perioperative vascular injury and preventing postoperative complications (including abdominal infection, gastrointestinal fistula) are the most important measures for preventing pseudoaneurysm formation, which can ultimately result in arteriointestinal fistula in GC patients after radical gastrectomy.

## Data Availability

Data sharing is not applicable to this article as no datasets were generated or analyzed during the current study.

## Abbreviations

GC: Gastric cancer
DSA: Digital subtraction angiography
GDA: Gastroduodenal artery
CHA: Common hepatic artery
SA: Splenic artery
PHA: Proper hepatic artery
RHA: Right hepatic artery
CT: Computed tomography
DG: Distal gastrectomy
TG: Total gastrectomy
R-Y: Roux-en-Y anastomosis
B II: Billroth II anastomosis
